# Dietary Aluminum Exposure Is More Closely Linked to Antioxidant Dynamics than to Body Mass Index

**DOI:** 10.3390/toxics13070578

**Published:** 2025-07-09

**Authors:** Ozge Yesildemir, Ceren Filiz Ozsoz, Mensure Nur Celik, Ozge Aydin Guclu, Anil Ozgur, Duygu Ağagündüz, Ferenc Budán

**Affiliations:** 1 Department of Nutrition and Dietetics, Faculty of Health Sciences, Bursa Uludag University, Bursa 16059, Türkiye; ozgeyesildemir@uludag.edu.tr; 2 Department of Food Engineering, Graduate School of Natural and Applied Sciences, Bursa Uludag University, Bursa 16285, Türkiye; ceren.cf@gmail.com; 3 Department of Nutrition and Dietetics, Faculty of Health Sciences, Ondokuz Mayıs University, Samsun 55200, Türkiye; mensurenur.celik@omu.edu.tr; 4 Department of Pulmonary Diseases, Faculty of Medicine, Bursa Uludag University, Bursa 16059, Türkiye; ozgeguclu@uludag.edu.tr (O.A.G.); anilozgur@uludag.edu.tr (A.O.); 5 Department of Nutrition and Dietetics, Faculty of Health Sciences, Gazi University, Ankara 06490, Türkiye; 6 Institute of Physiology, Medical School, University of Pécs, H-7624 Pécs, Hungary

**Keywords:** dietary aluminum, obesity, antioxidant capacity, environmental obesogen, diet quality

## Abstract

The association between aluminum exposure and obesity remains uncertain. This study investigated whether aluminum exposure (dietary, serum, and urinary) is linked to obesity and whether dietary antioxidant capacity moderates this relationship. A total of 54 adult women (26 obese, 28 normal weight) were recruited from a private weight loss clinic in Türkiye. Dietary aluminum exposure was estimated using 24 h dietary recalls and literature values, and antioxidant capacity was calculated through a food frequency questionnaire. Serum and spot urine samples were collected, and aluminum levels were measured using inductively coupled plasma optical emission spectrometry. No significant differences were observed between normal weight and obese groups in serum aluminum (127.7 ± 102.42 vs. 122.9 ± 88.37 µg/L, *p* > 0.05), urinary aluminum (28.1 ± 12.73 vs. 14.1 ± 10.77 µg/L, *p* > 0.05), or weekly dietary aluminum exposure (0.61 ± 0.45 vs. 0.45 ± 0.24 mg/kg bw/week, *p* > 0.05). Dietary aluminum exposure correlated positively with total antioxidant capacity (*r* = 0.665, *p* < 0.001). Regression analysis revealed that dietary aluminum exposure was inversely associated with body mass index (*β* = −0.27, *p* < 0.05), while antioxidant capacity did not moderate this relationship, nor did the age difference. These results suggest dietary aluminum exposure reflects diet quality and/or food preparation methods, etc., rather than directly influencing obesity.

## 1. Introduction

Obesity has become one of the most critical public health challenges of the 21st century [1]. According to the World Health Organization, in 2022, approximately one in eight people worldwide were living with obesity, with the prevalence of adult obesity more than doubling since 1990 [2]. Obesity is not only associated with excessive body weight but also plays a central role in the development of chronic diseases such as type 2 diabetes, cardiovascular conditions, and hypertension [3]. While traditionally explained as a simple imbalance between energy intake and expenditure, our understanding of obesity has evolved to encompass a more complex interplay of genetic, behavioral, metabolic, and environmental factors [4]. In this context, increased attention has been directed towards environmental chemicals that may contribute to obesity risk [5].

Ageing increases the likelihood of obesity and is associated positively with the accumulation of aluminum and toxic heavy metals (such as lead, cadmium, chromium, and arsenic) in the bones [6,7,8,9]. There is growing evidence that aging, obesity, and toxic metal accumulation—including aluminum—are interrelated, forming a pathophysiological triangle that amplifies risks for metabolic, cardiovascular, and neurodegenerative diseases, and many types of cancer [10,11].

Among these chemicals, aluminum has drawn significant attention due to its toxicological effects [12,13]. Although naturally abundant, aluminum is not an essential trace element for humans and has no recognized biological function [14]. Aluminum exposure can occur through various sources, including food additives, processed foods, packaging materials, cookware, drinking water, pharmaceuticals, and cosmetics, potentially leading to bioaccumulation and systemic toxicity [15,16]. The accumulation of aluminum in the body has been associated with the development of conditions such as neurodegenerative diseases, skeletal disorders, kidney damage, hormonal imbalances, and certain cancers [10].

Aluminum has been suggested to play a role as an environmental obesogen through several mechanisms. These include promoting intracellular lipid accumulation via mitochondrial dysfunction [17], impairing lipid metabolism through disruption of the carnitine shuttle and reduced β-oxidation [18], and altering eating behavior by disrupting the structure and signaling of peptide YY and neuropeptide Y [19]. However, direct evidence linking aluminum exposure to obesity remains limited. Several studies have reported significantly elevated aluminum concentrations in biological matrices such as hair and urine among overweight or obese patients [12,20,21]. Nevertheless, the extent to which dietary aluminum exposure relates to aluminum concentrations in these matrices and how far these levels differ by obesity status remains unclear. This highlights the need for more human-based data to test the environmental obesogen hypothesis.

One plausible pathway through which aluminum may contribute to obesity is via oxidative stress. Aluminum has been shown to impair intracellular antioxidant defense systems by enhancing the production of reactive oxygen species (ROS), which subsequently disrupts metabolic homeostasis [22]. This may cause cellular damage and increase fat tissue deposition, contributing to obesity [23]. At this point, it can be hypothesized that food-derived antioxidants may play a protective role by mitigating metabolic disruptions induced by aluminum-related oxidative stress. However, this potential interaction has not yet been directly investigated in scientific studies. Accordingly, studies that concurrently assess aluminum exposure and dietary antioxidant capacity are warranted to address a critical gap in the current body of literature. Therefore, this study aimed to investigate the association between aluminum exposure—assessed via dietary intake, serum, and urinary levels—and obesity in women. Additionally, we explored whether total dietary antioxidant capacity plays a moderating role in this relationship. To this end, 54 adult women (26 obese, 28 normal weight) were assessed using dietary records, a food frequency questionnaire, and blood and urine samples to determine aluminum exposure and antioxidant intake.

## 2. Materials and Methods

### 2.1. Study Design and Participants

This observational cross-sectional study was conducted at a private weight loss clinic in Bursa, Türkiye, between January and June 2024. The required sample size was estimated using G*Power software (version 3.0.10; Heinrich Heine University, Düsseldorf, Germany), assuming a two-sided alpha level of 0.05, 80% power, and differences between independent means. A minimum of 25 participants per group was determined to be sufficient. Accordingly, a total of 54 women aged 19–65 years who visited the clinic during the study period were recruited. In order to reduce biological and behavioral variability associated with sex differences and to allow for a more homogeneous study population, only female participants were included in the study by design. Participants were categorized into two groups according to their body mass index (BMI): the obese group (BMI ≥ 30.0 kg/m^2^; n = 26) and the normal weight group (BMI 18.5–24.99 kg/m^2^; n = 28).

Exclusion criteria included current or former smoking, occupational exposure to environmental toxins (such as metal emissions), pregnancy or lactation, psychiatric disorders, gastrointestinal diseases treated with aluminum-containing antacids, presence of metal implants (including dental amalgams), use of mineral supplements, adherence to vegetarian or other non-standard diets, and residence near industrial emission sources. Volunteers who did not meet the exclusion criteria were included in the study.

The study was approved by the Bursa Uludag University Health Research Ethics Committee (approval number: 2024-3/1, date: 6 May 2024). All procedures were conducted according to the Declaration of Helsinki. Written informed consent was obtained from all participants.

### 2.2. Study Protocol and Data Collection

After enrollment and group assignment, participants completed a structured, interviewer-administered questionnaire that collected information on sociodemographic characteristics (age, education level, and income), health status, and dietary intake. Two dietary assessment tools were used: a 24 h dietary recall to estimate food and nutrient intake, and a food frequency questionnaire (FFQ) to estimate dietary antioxidant capacity. Anthropometric measurements were also taken. Blood and urine samples were collected for aluminum analysis.

#### 2.2.1. Anthropometric Measurements

Body weight was measured to the nearest 0.1 kg using a calibrated digital scale (Model 48495; Medisana GmbH, Neuss, Germany) with participants wearing light clothing and no shoes. Height was measured using a portable Leicester stadiometer (Marsden Group, Rotherham, UK) while participants stood upright, taking a deep breath, with their heads positioned in the Frankfort horizontal plane. BMI was calculated as weight in kilograms divided by the square of height in meters (kg/m^2^).

#### 2.2.2. Assessment of Dietary Aluminum Exposure

Dietary aluminum intake was estimated using the single daily 24 h dietary recall method. Portion sizes of consumed foods were determined using the “Photo Catalog of Foods and Nutrients: Measures and Amounts” [24]. Information regarding home-prepared meals was gathered by asking participants about the ingredients and preparation methods. Standardized recipes from the “Standard Recipes for Institutions Providing Mass Catering” book [25] were used to estimate food quantities for foods consumed outside the home. Daily intake of consumed foods was calculated using the BEBIS 9.0 (Nutrition Information System; Istanbul, Türkiye) program.

Participants’ weekly dietary aluminum exposure was estimated using the aluminum concentrations in foods, body weight, and the total amount of foods consumed per day. The amount of food consumed by individuals was determined using the 24 h dietary recall method, as mentioned above. However, no existing database was available for the aluminum content of commonly consumed foods in Türkiye. Aluminum content data were compiled from the existing literature [26,27,28,29,30], particularly from findings regarding aluminum content in foods common in Turkish cuisine. The following equation was used to evaluate the weekly exposure to aluminum from food consumption [31,32]:

$$Aluminum\;exposure=\frac{Mean\;concentration\;of\;aluminum\times Consumption\;amount\;of\;food\;}{body\;weight\;}\times \;7$$

where a unit of aluminum exposure = mg/kg bw/week, mean concentration of aluminum = mg/kg, consumption amount of food = kg/day, body weight = kg, and 7 = the conversion factor of day to week. This formula was applied to calculate the aluminum intake for each food item. Afterward, the aluminum exposures from all food items were summed to obtain the total weekly aluminum exposure for each individual.

#### 2.2.3. Assessment of Dietary Antioxidant Capacity

Total dietary antioxidant capacity was estimated using an FFQ adapted from Satia et al. [33] and modified for Turkish dietary habits. The FFQ included major food groups such as fruits, vegetables, cereals–nuts–snacks, meat–eggs–dairy products, mixed dishes–soups, sauces–condiments–fats, and beverages, each comprising items known to be rich in antioxidants. Participants reported their consumption frequency and portion sizes for each item over the past month. Frequency options included “2 or more times a day”, “once a day”, “5–6 times a week”, “3–4 times a week”, “1–2 times a week”, “2–3 times a month”, “once a month”, and “never”. Portion sizes (in grams or milliliters) were recorded for each item. The “Photo Catalog of Foods and Nutrients: Measures and Amounts” [24] was used to estimate portion sizes. Based on the reported frequency and portion sizes via the antioxidant food frequency questionnaire, the average daily intakes of antioxidant-rich foods were calculated. The antioxidant content of the consumed foods was also analyzed using the BeBiS 9.0 (Nutrition Information System) program. The BeBiS database includes antioxidant values derived from the Antioxidant Food Table developed by Carlsen et al. (2010), which reports the total antioxidant capacity of over 3100 food and beverage items based on the Ferric Reducing Ability of Plasma (FRAP) assay [34]. Dietary supplements were not included in the dietary antioxidant capacity calculation due to a lack of detailed consumption data.

#### 2.2.4. Biological Sample Collection

After dietary assessments, venous blood (5 mL) was drawn from the antecubital vein into trace-element-free tubes by trained personnel. Spot urine samples (50 mL) were collected in clean, sterile containers. Blood and urine samples were collected in the morning (08:00–10:00) during fasting. This timing was chosen to minimize possible fluctuations in biomarker levels during the day and to provide a standard sampling time. Blood samples were centrifuged at 3000 rpm for 10 min to separate serum, which was aliquoted into Eppendorf tubes. All samples were immediately stored at −80 °C until analysis.

#### 2.2.5. Aluminum Analysis

For urine analysis, 5 mL of urine was mixed with 3 mL of 65% nitric acid (HNO_3_) and 2 mL of 30% hydrogen peroxide (H_2_O_2_). The mixture was vortexed thoroughly to ensure proper mixing of the chemicals. For serum analysis, 0.5 mL of serum was combined with 3 mL of 65% HNO_3_ and 1.5 mL of 30% H_2_O_2_. The mixture was vortexed to ensure complete homogenization of the reagents. Each sample was filtered on Whatman grade 42 paper (Cytiva, Marlborough, MA, USA) graduated to 50 mL with deionized water and refrigerated at 3 °C until its analysis. Then, the samples were introduced into an inductively coupled plasma optical emission spectrometer (ICP-OES; Model Optima 8000; PerkinElmer Inc., Waltham, MA, USA) for analysis. The ICP-OES system was calibrated using blank and standard solutions, and aluminum emission lines were measured for both urine and serum samples. The analysis was conducted under optimized conditions, and calibration was verified periodically by analyzing standard solutions every 10 readings to ensure the accuracy and precision of the results. The coefficient of determination (R^2^) for the calibration curves was found to be 0.9997 for aluminum, indicating excellent linearity. Limit of detection (LOD) and limit of quantification (LOQ) were obtained at 0.035 µg/L and 0.106 µg/L, respectively.

### 2.3. Statistical Analysis

Statistical analysis was performed using the Statistical Package for the Social Sciences (SPSS) Version 28 (IBM Inc., Armonk, NY). Numerical variables were expressed as arithmetic mean (mean), standard deviation (SD), median (M), and interquartile range (IQR). Categorical variables were expressed as frequencies (n) and percentages (%). The Shapiro–Wilk test was used to assess the normality of the distribution of variables. For comparisons between two independent groups, the Independent Sample *t*-test was applied for normally distributed variables, and the Mann–Whitney U test was used for non-normally distributed variables. Differences between categorical variables were evaluated using the Chi-square test. The Pearson correlation coefficient was used to examine the relationship between two numerical variables with normal distribution, while the Spearman correlation coefficient was used for non-normally distributed variables. Correlation matrices were visualized using heatmaps generated in Python (version 3.10.12; Python Software Foundation, Wilmington, DE, USA), with statistically significant correlations (*p* < 0.05) marked by asterisks. Multiple linear regression models were used to identify factors associated with BMI. Variables were added hierarchically in five models, including demographics, aluminum biomarkers, dietary aluminum exposure, antioxidant capacity, and their interaction. Statistical significance was set at a *p*-value of less than 0.05.

## 3. Results

The median age was significantly higher in the obese group compared with the normal weight group (45.0 vs. 31.5 years, *p* < 0.05). A significantly greater proportion of participants in the obese group were married (76.9%) compared with the normal weight group (35.7%) (*p* < 0.05). No statistically significant differences between the groups regarding education level, income level, or disease status were found (*p* > 0.05). Body weight and BMI were significantly higher in the obese group compared with the normal weight group (*p* < 0.05) (Table 1).

The mean serum aluminum levels were 127.7 ± 102.42 µg/L in the normal weight group and 122.9 ± 88.37 µg/L in the obese group, with no statistically significant difference (*p* > 0.05). Similarly, urinary aluminum levels were lower in the obese group (14.1 ± 10.77 µg/L) compared with the normal weight group (28.1 ± 12.73 µg/L), and the difference was not statistically significant (*p* > 0.05). Weekly dietary aluminum exposure was also comparable, with mean values of 0.61 ± 0.45 mg/kg bw/week in the normal weight group and 0.45 ± 0.24 mg/kg bw/week in the obese group (*p* > 0.05) (Table 2).

**Figure 1:**
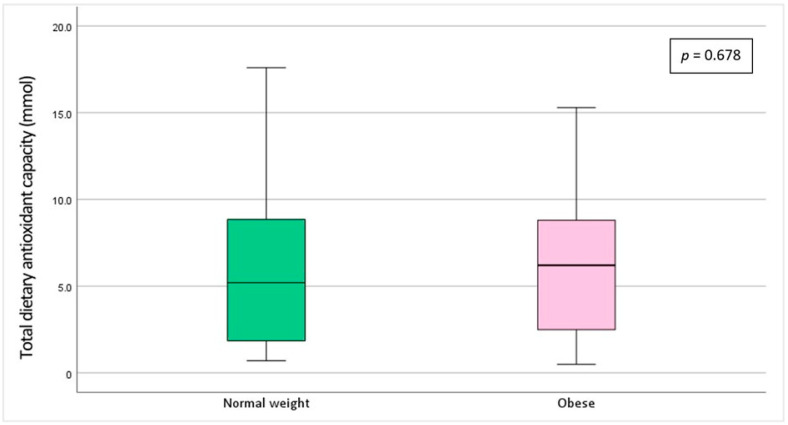
Total dietary antioxidant capacity across body mass index categories. Each boxplot displays the median, interquartile range (25th–75th percentiles), and full data range (minimum to maximum). Outliers were omitted to enhance clarity.

Total dietary antioxidant capacity did not significantly differ between groups (6.2 ± 4.97 mmol in the normal weight group vs. 6.4 ± 4.52 mmol in the obese group, *p* > 0.05) (Figure 1).

**Figure 2:**
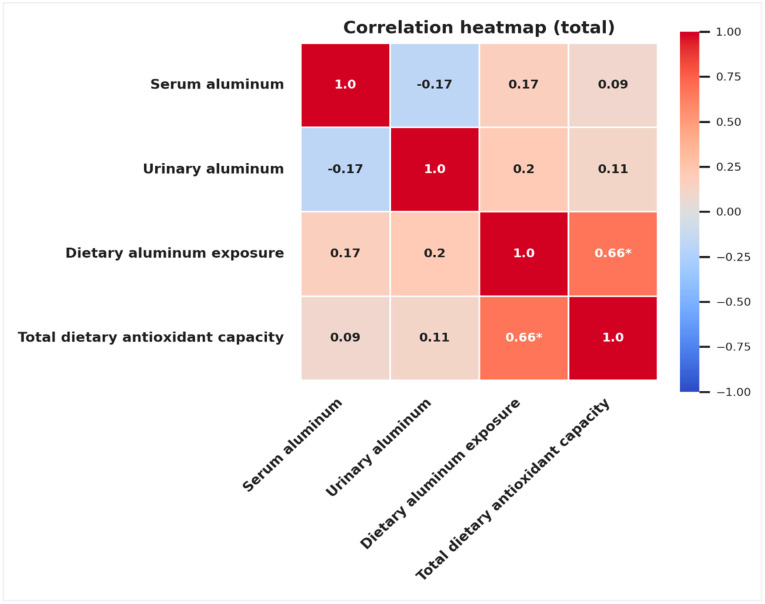
Pearson correlation heatmap for analysis between serum and urinary aluminum levels, dietary aluminum exposure, and total dietary antioxidant capacity in all participants. Positive correlations are shown in red and negative correlations in blue. The color intensity represents the strength of the correlation. Significant correlations at *p* < 0.01 are marked with an asterisk.

Dietary aluminum exposure was significantly and positively correlated with total dietary antioxidant capacity in all participants (*r* = 0.665, *p* < 0.001). This relationship remained significant within both BMI subgroups, with a stronger correlation observed among normal weight individuals (*r* = 0.798, *p* < 0.001) compared with those with obesity (*r* = 0.500, *p* < 0.05). In contrast, no statistically significant correlations were observed between serum or urinary aluminum levels and total dietary antioxidant capacity in any group (*p* > 0.05) (Table 3, Figure 2). Subgroup-specific patterns are further illustrated in Supplementary Figures S1 and S2.

Regression models were constructed to identify potential factors affecting individuals’ BMI. In Model 1, age, education, and income were included. Age was positively and significantly associated with BMI, indicating that each one-year increase in age was associated with a 0.49-unit increase in BMI (*p* < 0.001), holding other variables constant. Education and income levels were not significant predictors (*p* > 0.05). This model explained 26.4% of the variance in BMI (Adjusted R^2^ = 0.264). In Model 2, serum and urinary aluminum levels were added. Age remained a significant predictor (*p* < 0.001), while neither serum nor urinary aluminum levels showed a significant association with BMI (*p* > 0.05). The explained variance slightly decreased (Adjusted R^2^ = 0.258). In Model 3, dietary aluminum exposure was included. A one-unit increase in dietary aluminum exposure was related to a 0.27-unit decrease in BMI (*p* < 0.05). Age remained a significant predictor (*p* < 0.001). This model showed the highest explanatory power (Adjusted R^2^ = 0.314). In Model 4, total dietary antioxidant capacity was added to the model. This variable was not significantly associated with BMI (*p* > 0.05), and the model fit slightly declined (Adjusted R^2^ = 0.299). Model 5 included an interaction term between dietary aluminum exposure and antioxidant capacity to test for moderation. The interaction was not statistically significant (*p* > 0.05), indicating that antioxidant capacity did not moderate the relationship between dietary aluminum exposure and BMI. The final model explained 28.4% of the variance (Adjusted R^2^ = 0.284) (Table 4).

## 4. Discussion

To our knowledge, this is the first study to simultaneously evaluate dietary aluminum exposure, biological aluminum indicators (serum and urine levels), and total dietary antioxidant capacity in relation to obesity or age, respectively. This integrated approach provides a novel perspective on the potential influence of dietary exposure on body weight regulation. In our findings, serum and urinary aluminum concentrations did not significantly differ between normal weight and obese groups. Likewise, dietary aluminum exposure and antioxidant intake were similar across BMI groups. However, dietary aluminum exposure showed a strong positive correlation with antioxidant capacity, particularly among normal weight females. Multiple regression analyses indicated an inverse association between dietary aluminum exposure and BMI, while antioxidant capacity had no independent effect and did not moderate this relationship. These results underscore the need for more holistic models that consider both environmental exposures and dietary patterns in the context of obesity research.

The literature on the relationship between aluminum exposure and obesity is limited and presents inconsistent findings. Some studies have reported higher aluminum levels in obese individuals compared with lean controls. For example, elevated aluminum concentrations have been observed in hair and urine samples of obese individuals without occupational exposure, with urinary aluminum levels showing a positive correlation with BMI [12]. Similarly, another study reported increased hair aluminum levels in obese subjects [21]. In contrast to these results, our study did not observe significant differences in serum and urine aluminum levels between normal weight and obese participants. This discrepancy may reflect the complex biological mechanisms underlying metal metabolism in the context of obesity. Chronic low-grade inflammation—a hallmark of obesity—can modulate the expression and function of metal transporters [35,36]. One such transporter, divalent metal transporter 1 (DMT1), primarily facilitates iron absorption but may also mediate the uptake of other trivalent metals, including aluminum, due to its limited substrate specificity under certain physiological conditions [37]. According to the observations of Cannata et al. in humans, ferritin levels (which determine iron status) are inversely correlated to the absorption of aluminum [38].

Elevated inflammatory cytokines associated with obesity can influence the expression of DMT1 and other metal transporters, suggesting a direct link between inflammation and altered metal absorption, thereby affecting aluminum kinetics [39]. Thus, the inflammatory state in obesity may lead to changes in aluminum absorption, distribution, or excretion. These inflammation-driven alterations in aluminum handling may account for the inconsistencies observed in the literature. Overall, these findings imply that BMI alone may not sufficiently capture the metabolic and inflammatory heterogeneity that modulates aluminum metabolism in the body.

Estimating daily and weekly aluminum intake is an important component of risk assessment and regulatory evaluations of this health-harming toxic metal. In 2011, the Joint Expert Committee on Food Additives (JECFA) recommended a Provisional Tolerable Weekly Intake (PTWI) of 2 mg/kg bw/week [40]. The European Food Safety Authority (EFSA) recommended 1 mg/kg bw/week [41]. Aluminum exposure varies considerably between countries/regions. For example, individuals aged 20–50 years living in Shenzhen, southern China, show exposure levels around 1.26 mg/kg bw/week [42], whereas those in Tianjin, northern China, have been reported to reach as high as 8.40 mg/kg bw/week [43]. In Europe, exposure is generally lower, with estimates of 0.75 mg/kg bw/week in Greece [26], 0.48 mg/kg bw/week in Italy [44], 0.18–0.21 mg/kg bw/week in Germany [45], and as low as 0.030 mg/kg bw/week in Belgium [46]. In the present study, dietary aluminum intake per kilogram body weight in both the normal weight and obese groups remained below the established safety thresholds, aligning with values observed in most European populations. Although the mean exposure values between the groups did not differ significantly, minor differences may be attributable to variations in cooking practices, food consumption patterns, and the use of aluminum-containing additives or kitchenware. For instance, it is well documented that the use of aluminum cookware and storage containers can substantially elevate the aluminum content of food [47,48]. Since the use of equipment was not questioned in this study, it cannot be determined with certainty whether the differences between the groups were due to kitchen equipment. Taken together, while overall exposure levels appear to be within acceptable safety margins, our findings underscore the need to consider both dietary sources and non-dietary determinants of aluminum exposure, such as cookware and food packaging, in future research.

Diet is the most important source of aluminum exposure for a population without occupational exposure [49,50,51]. In this study, dietary aluminum exposure had a significant positive correlation with total dietary antioxidant capacity in all participants, with stronger correlations in normal weight individuals compared with obese individuals. This may be partly explained by the contribution of plant-based foods—such as tea, leafy greens, grains, and herbs—that are both high in antioxidants and known to contain aluminum [34]. Therefore, dietary aluminum intake may serve not only as an indicator of potential metal exposure but also as a proxy for particular dietary patterns. Although mean aluminum and antioxidant intakes did not significantly differ between BMI groups, variations in food choices within these groups may account for the observed difference in correlation strength. Furthermore, the absence of a significant correlation between dietary antioxidant capacity and serum or urinary aluminum levels aligns with the literature reporting the low and variable bioavailability of dietary aluminum [52,53]. Thus, dietary aluminum intake may indicate food selection rather than actual systemic exposure. This pattern may also suggest that individuals with obesity could be more vulnerable to aluminum toxicity, not merely due to higher exposure, but possibly due to impaired nutrient status. Additionally, the lack of association between antioxidant intake and aluminum biomarkers suggests that the protective role of antioxidants against aluminum accumulation or toxicity may be limited or dependent on other factors such as timing, bioavailability, or interaction with inflammatory pathways. This finding emphasizes the need for further mechanistic studies investigating how antioxidant status influences aluminum metabolism and toxicity, especially in the context of obesity-related oxidative stress.

Serum and urine aluminum levels were not significantly associated with BMI. However, dietary aluminum intake showed a significant negative association with BMI, which appears contrary to our initial hypothesis that higher aluminum exposure might relate to increased obesity risk. This unexpected finding may be explained by the low energy density of some aluminum-rich foods. For instance, one study reported higher aluminum concentrations in vegetables, fruits, and seafood compared with other food groups [54]. Aluminum levels can be high, especially in vegetables, due to aluminum absorption and accumulation from the soil [55]. EFSA also states that vegetables, cereals, and beverages can be major sources of aluminum intake [56]. Nevertheless, the contribution of different food groups to total aluminum exposure depends not only on aluminum content but also on the frequency of consumption. Therefore, future studies focusing on detailed assessments of aluminum intake by food groups, alongside consumption patterns, would be valuable to clarify this relationship.

Furthermore, ageing and exposure to aluminum (or heavy metals such as lead, arsenic, and cadmium), as well as obesity, can cause dysbiosis. Meanwhile, dysbiosis harms essential mineral uptake in the gut by damaging tight junctions [57,58,59]. This could lead theoretically to a self-sustaining circle, which is worsened by the inflammatory signal transducers, forming a pathophysiological quadrilateral system. However, synbiotic supplementation can treat obesity successfully by curing dysbiosis as the first step [60]. If the members of the obese group had received synbiotic food supplements as part of the private weight loss clinic’s therapeutic approach, while the younger and normal weighted group members did not, this could explain the contradictory results of this study. However, the proposed role of dysbiosis as a mediating mechanism remains hypothetical in the absence of direct assessments such as gut microbiota profiling or intestinal permeability markers. Therefore, the interpretation of this pathway should be approached with caution. Future studies may need to include individuals using synbiotic and similar supplements in their exclusion criteria in order to control for their effects.

Preparing and storing food and drinks can have a very high impact on aluminum content. Fruit juices contain from 49.3 to 1144.6 μg/L and soft drinks from 44.6 to 1053.3 μg/L aluminum [61]. The consumption of fruit juices, cola, and energy drinks with a high sugar content is especially popular among adolescents and young adults in Türkiye [62]. However, storing them in an aluminum can for days at room temperature may multiply the aluminum content of foods and soft drinks [63]. Acidic conditions may multiply the concentration of soluble aluminum too [63]. For example, pickles or chutneys in an aluminum vessel at room temperature may contain 100 mg/kg aluminum [63].

This study has some limitations. Firstly, information on food preparation, cooking, and storage practices—including the use of aluminum-containing cookware, packaging, or containers for acidic foods—was not collected, which may have led to an underestimation of total aluminum exposure. Future studies should incorporate detailed assessments of non-dietary aluminum sources, including cookware and storage materials, to provide a more comprehensive evaluation of total exposure. Secondly, dietary aluminum content data were obtained from the existing literature rather than direct food analysis, which may reduce the precision of exposure assessment. Further studies should consider direct measurement of aluminum content in locally consumed foods to enhance exposure accuracy. Also, the use of an FFQ inherently relies on participants’ memory and is subject to recall bias, as individuals were asked to report their consumption over the past month. Additionally, seasonal dietary variations were not considered, and consumption was averaged across a one-month period, potentially limiting the accuracy of exposure estimates. Also, the single daily 24 h dietary recall used in this study may pose a potential limitation in assessing dietary aluminum exposure, as it may not fully reflect the habitual dietary patterns of individuals. Another limitation is that dietary supplements were not included due to insufficient usage data, which may have resulted in a lower estimate of overall antioxidant intake. Moreover, the antioxidant values in the BeBiS database may not reflect the most recent compositional changes in food items. The FRAP method employed in this study assesses only the reducing capacity of antioxidants and does not capture their full biological activity, including reactive oxygen species scavenging and the modulation of antioxidant enzymes [64]. In addition, although the sample size was determined a priori using power analysis and deemed sufficient to detect statistically meaningful differences between groups, the relatively small number of participants may still limit the generalizability and robustness of the findings. Furthermore, the study was conducted exclusively among women, which enhanced internal consistency but restricted the extrapolation of the results to male populations. Later research should replicate these findings in larger and more diverse cohorts to strengthen external validity. It should also be noted that the observed positive correlation between dietary aluminum exposure and antioxidant capacity likely reflects the consumption of plant-rich diets. However, the relative contributions of specific food groups, such as tea and vegetables, to this association were not dissected in this study. Upcoming studies should explore these individual food group effects to better understand the sources of aluminum and antioxidants in the diet. Lastly, although dysbiosis was discussed as a potential mechanistic link between aluminum exposure and obesity, our study did not include microbiome analyses or any relevant biomarkers. Future mechanistic studies should incorporate such measurements to better elucidate the underlying pathways. In summary, while our study thoroughly discusses the main limitations, including exposure assessment gaps, mechanistic pathways, and sample generalizability, addressing these complex issues requires further dedicated research beyond the scope of the current work. We believe our findings provide valuable preliminary insights and a foundation for such future investigations.

## 5. Conclusions

The relationship between aluminum exposure and obesity remains unclear and is likely influenced by both dietary intake and accumulated aluminum levels in the body. In this study, no significant associations were observed between serum or urinary aluminum concentrations and BMI or dietary antioxidant capacity. In contrast, dietary aluminum intake was significantly associated with total dietary antioxidant capacity, particularly among normal weight individuals. This finding suggests that dietary aluminum intake may reflect overall diet quality rather than serve as a direct contributor to obesity. Further research is needed to better understand these complex interactions and to explore the potential role of dietary patterns and food sources in mediating the effects of aluminum exposure on health outcomes.

## Figures and Tables

**Table 1:** Sociodemographic characteristics of the participants.

Variables	Total (n = 54)	Normal Weight (n = 28)	Obese (n = 26)	*p*
Age (years), M (IQR)	41.0 (25.75)	31.5 (27.75)	45.0 (13.62)	**0.004**
Education level, n (%)				
Primary school	5 (9.3)	0 (0)	5 (9.3)	0.840
High school	9 (16.7)	4 (14.3)	9 (16.7)	
University	34 (63.0)	20 (71.4)	34 (63.0)	
Post-graduate	6 (11.0)	4 (14.3)	6 (11.0)	
Marital status, n (%)				
Married	30 (55.6)	10 (35.7)	20 (76.9)	**0.003**
Single	24 (44.4)	18 (64.3)	6 (23.1)	
Income level, n (%)				
Low	6 (11.1)	2 (7.1)	4 (15.4)	0.613
Medium	33 (61.1)	17 (60.7)	16 (61.5)	
High	15 (27.8)	9 (32.2)	6 (23.1)	
Disease status, n (%)				
With chronic disease	18 (33.3)	7 (25.0)	11 (42.3)	0.145
Without chronic disease	36 (66.7)	21 (75.0)	15 (57.7)	
Body weight (kg), mean ± SD	74.3 ± 15.97	61.8 ± 7.77	87.8 ± 10.51	**<0.001**
Body mass index (kg/m^2^), mean ± SD	27.8 ± 6.17	22.6 ± 1.94	33.3 ± 3.76	**<0.001**

Normal variables were presented as mean and standard deviation (Mean ± SD), and non-normal variables were presented as the median and interquartile range (M (IQR)). The Sample *t*-test and the Mann–Whitney U test were applied for normally and non-normally distributed variables, respectively. The Chi-square test was used to evaluate the differences between categorical variables. The bold values indicate statistically significant values (*p* < 0.05).

**Table 2:** Comparison of serum and urinary aluminum levels and dietary aluminum exposure between the normal weight group and the group of obese participants.

Variables	Normal Weight (n = 28)		Obese (n = 26)		*p*
	Mean ± SD	Min–Max	Mean ± SD	Min–Max	
Serum aluminum (µg/L)	127.7 ± 102.42	10.9–346.4	122.9 ± 88.37	9.9–299.6	0.449
Urinary aluminum (µg/L)	28.1 ± 12.73	<LOD-367.1	14.1 ± 10.77	1.98–41.6	0.165
Dietary aluminum exposure (mg/kg bw/week)	0.61 ± 0.45	0.16–1.65	0.45 ± 0.24	0.04–0.95	0.563

LOD: Limit of detection; SD: Standard deviation; Min: Minimum; Max: Maximum; bw: Body weight. For comparisons between two independent groups, the Independent Sample *t*-test was applied.

**Table 3:** Correlation between dietary antioxidant capacity, serum and urinary aluminum levels, and dietary aluminum exposure.

	Total Dietary Antioxidant Capacity					
	Total (n = 54)		Normal Weight (n = 28)		Obese (n = 26)	
	*r*	*p*	*r*	*p*	*r*	*p*
Serum aluminum (µg/L)	0.086	0.539	0.222	0.256	−0.101	0.624
Urinary aluminum (µg/L)	0.114	0.413	0.130	0.509	0.210	0.303
Dietary aluminum exposure (mg/kg bw/week)	0.665	**<0.001**	0.798	**<0.001**	0.500	**0.009**

*r*: Pearson correlation coefficient. bw: Body weight. The bold values indicate statistically significant values (*p* < 0.05).

**Table 4:** Regression models examining potential factors associated with body mass index.

	Model 1			Model 2			Model 3			Model 4			Model 5		
	*β*	*se*	*p*	*β*	*se*	*p*	*β*	*se*	*p*	*β*	*se*	*p*	*β*	*se*	*p*
Age	0.49	0.048	**<0.001**	0.473	0.049	**<0.001**	0.514	0.048	**<0.001**	0.511	0.049	**<0.001**	0.504	0.053	**<0.001**
Education level	−0.190	0.981	0.130	−0.170	0.995	0.181	−0.154	0.958	0.208	−0.151	0.990	0.232	−0.147	1.016	0.255
Income level	−0.096	1.218	0.426	−0.107	1.228	0.382	−0.070	1.192	0.556	−0.070	1.208	0.556	−0.073	1.234	0.549
Serum aluminum				−0.011	0.008	0.925	0.045	0.008	0.707	0.045	0.008	0.708	0.046	0.008	0.704
Urinary aluminum				−0.152	0.015	0.220	−0.091	0.015	0.455	−0.090	0.015	0.463	−0.091	0.015	0.465
Dietary aluminum exposure							−0.271	2.018	**0.031**	−0.284	2.704	0.089	−0.242	5.146	0.440
Total dietary antioxidant capacity										0.019	0.213	0.906	0.054	0.356	0.845
Interaction: Dietary Aluminum × Antioxidant Capacity													−0.070	0.485	0.875
Adjusted R^2^	0.264			0.258			0.314			0.299			0.284		

The bold values indicate statistically significant values (*p* < 0.05). Age is measured in years. Serum aluminum and urinary aluminum are measured in µg/L. Dietary aluminum exposure is measured in mg/kg bw/week. Total dietary antioxidant capacity is measured in mmol. Variable values: Education level (Primary school graduate = 1, High school graduate = 2, University graduate = 3, Post-graduate = 4), Income level (Low = 1, Medium = 2, High = 3).

## Data Availability

The data presented in this study is available on request from the corresponding author. The data is not publicly available due to privacy.

## Supplementary Materials

The following supporting information can be downloaded at: https://www.mdpi.com/article/10.3390/toxics13070578/s1, Figure S1: Pearson correlation heatmap for analysis between serum and urinary aluminum levels, dietary aluminum exposure, and total dietary antioxidant capacity in normal weight participants; Figure S2: Pearson correlation heatmap for analysis between serum and urinary aluminum levels, dietary aluminum exposure, and total dietary antioxidant capacity in obese participants.

## Abbreviations

The following abbreviations are used in this manuscript:

BMI	Body mass index
BW	Body weight
FFQ	Food frequency questionnaire
ICP-OES	Inductively coupled plasma optical emission spectrometer
IQR	Interquartile range
LOD	Limit of detection
M	Median
Max	Maximum
Min	Minimum
ROS	Reactive oxygen species
SD	Standard deviation
