# Supplementary figures and images for: Dietary Aluminum Exposure Is More Closely Linked to Antioxidant Dynamics than to Body Mass Index
Source: Toxics. 2025 Jul 9;13(7):578. doi: 10.3390/toxics13070578 (PMC12300108; doi:10.3390/toxics13070578)

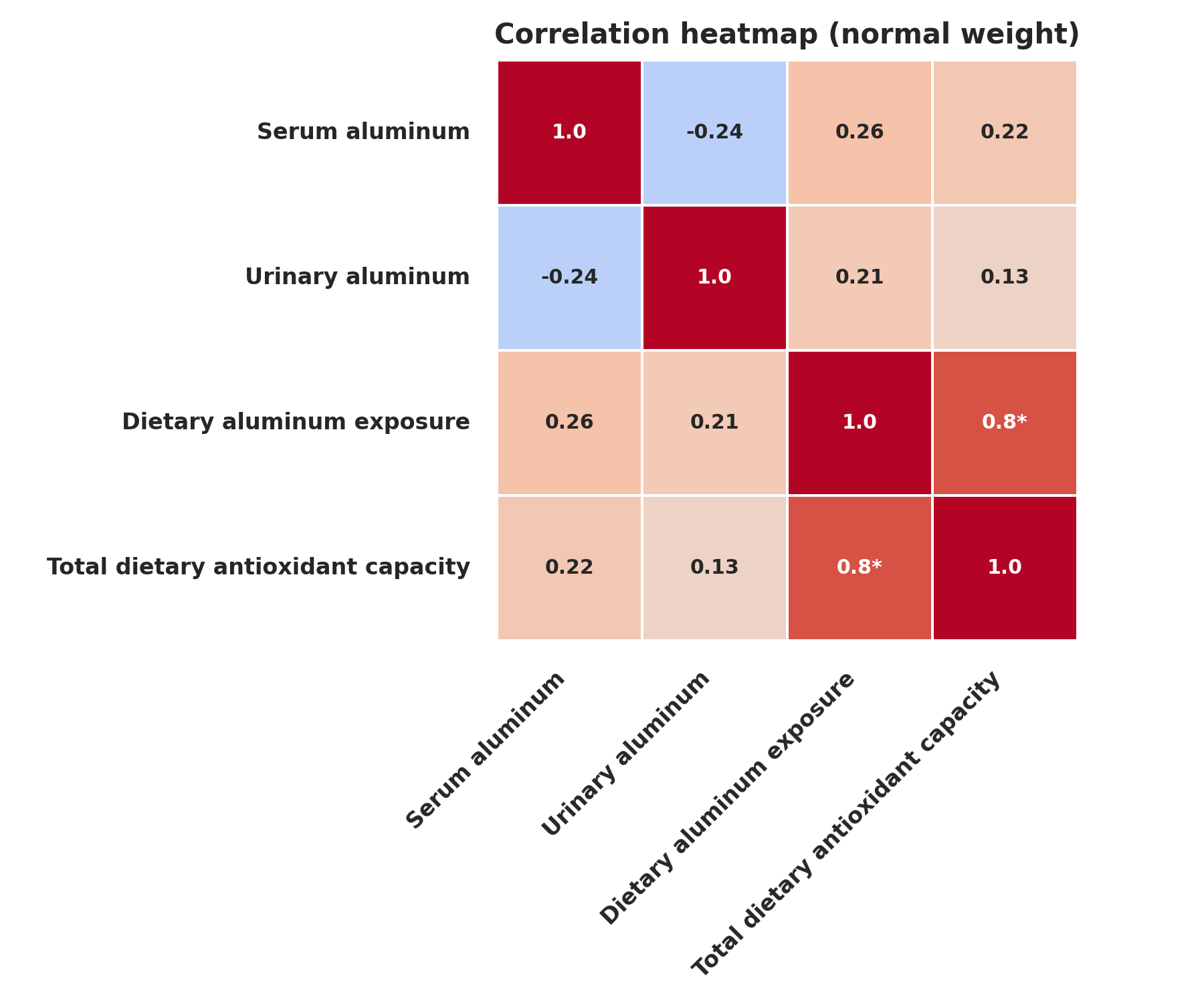

Supplement: Supplementary file 1 [file toxics-13-00578-s001.zip › supplementary files/Supp Figure 1.png]

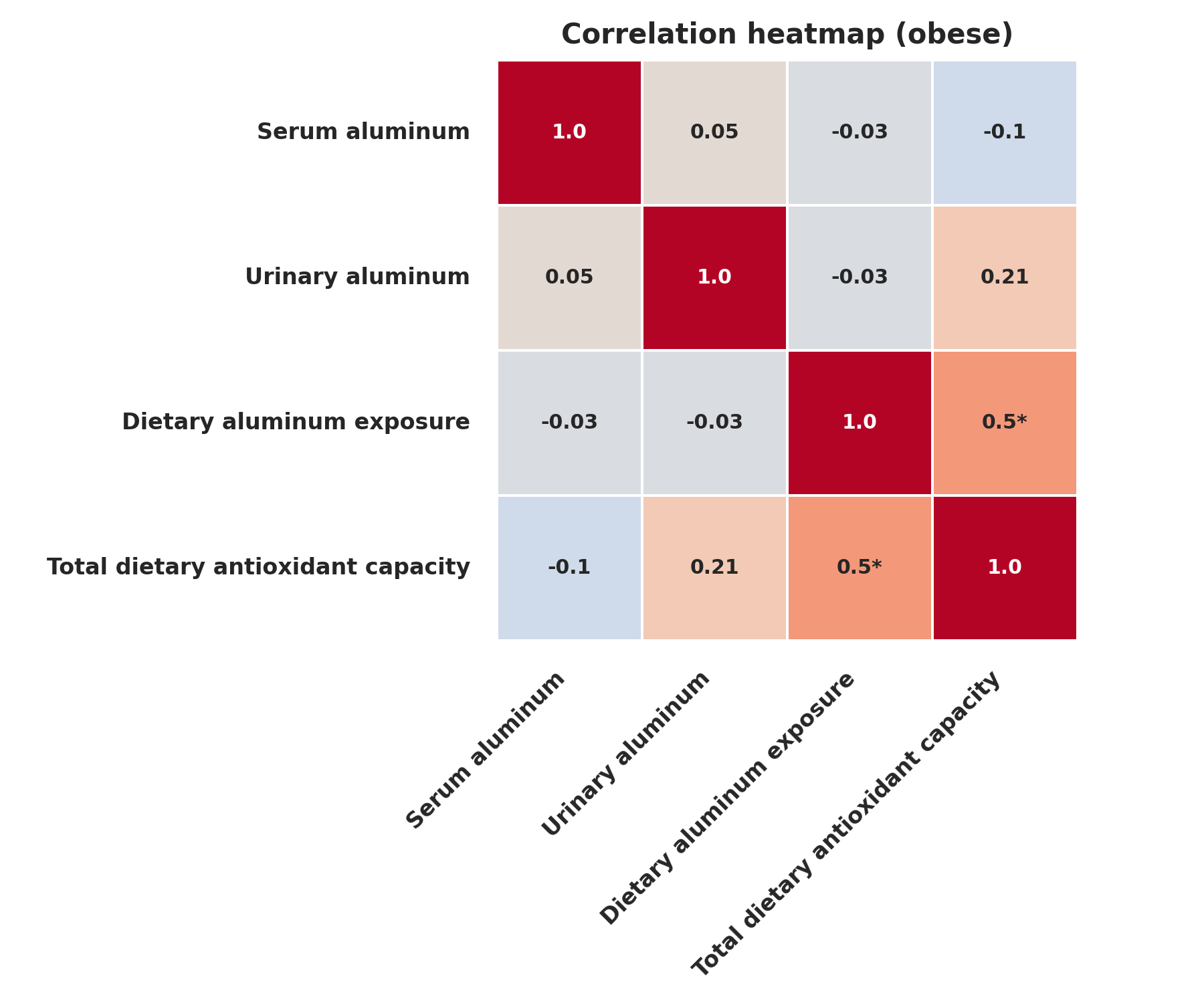

Supplement: Supplementary file 1 [file toxics-13-00578-s001.zip › supplementary files/Supp Figure 2.png]
